# Emerging Roles of Disabled Homolog 2 (DAB2) in Immune Regulation

**DOI:** 10.3389/fimmu.2020.580302

**Published:** 2020-10-15

**Authors:** Vanessa Figliuolo da Paz, Fayez K. Ghishan, Pawel R. Kiela

**Affiliations:** ^1^ Department of Pediatrics, University of Arizona, Tucson, AZ, United States; ^2^ Department of Immunobiology, University of Arizona, Tucson, AZ, United States

**Keywords:** Dab2, immune cells, immunoregulation, inflammation, macrophage, dendritic cells, lymphocytes

## Abstract

Disabled-2 (DAB2) is a clathrin and cargo binding endocytic adaptor protein recognized for its multifaceted roles in signaling pathways involved in cellular differentiation, proliferation, migration, tumor suppression, and other fundamental homeostatic cellular mechanisms. The requirement for DAB2 in the canonical TGFβ signaling in fibroblasts suggested that a similar mechanism may exist in immune cells and that DAB2 may contribute to immunological tolerance and suppression of inflammatory responses. In this review, we synthesize the current state of knowledge on the roles of DAB2 in the cells of the innate and adaptive immune system, with particular focus on antigen presenting cells (APCs; macrophages and dendritic cells) and regulatory T cells (Tregs). The emerging role of DAB2 in the immune system is that of an immunoregulatory molecule with significant roles in Treg-mediated immunosuppression, and suppression of TLR signaling in APC. DAB2 itself is downregulated by inflammatory stimuli, an event that likely contributes to the immunogenic function of APC. However, contrary findings have been described in neuroinflammatory disorders, thus suggesting a highly context-specific roles for DAB2 in immune cell regulation. There is need for better understanding of DAB2 regulation and its roles in different immune cells, their specialized sub-populations, and their responses under specific inflammatory conditions.

## Introduction

**Figure 1:**
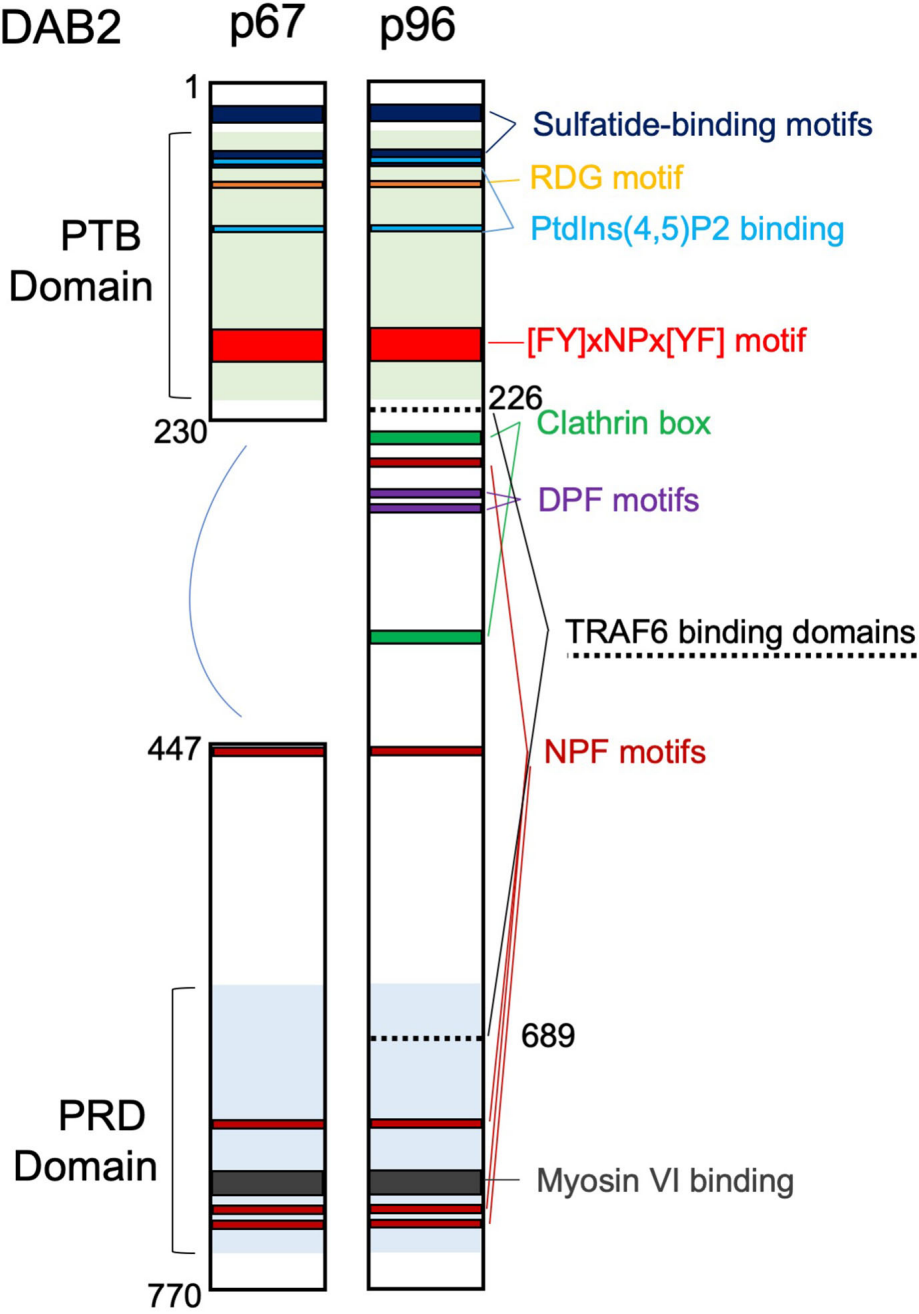
Domain structure and main binding motifs of the two Disabled‐2 (Dab2) isoforms (p96 and p67). PTB, phosphotyrosine‐binding domain; PRD, proline‐rich domain; Motifs: RGD (Arg‐Gly‐Asp); [FY]xNPx[YF], ([Phe or Tyr]‐any‐Asn‐Pro‐any‐[Tyr or Phe]); DPF, Asp‐Pro‐Phe; NPF, Asn‐Pro‐Phe. TRAF6 binding domains are also indicated.

Disabled homolog 2 (DAB2) received its name due to a high degree of similarity to the amino-terminal of the Disabled (Dab) protein initially described in *Drosophila melanogaster* as involved in embryonic neural development. Unbeknownst of its homology to Dab, in 1994 Mok et al. (1) described the partial cDNA of a gene with severely reduced expression in ovarian cancer and named it DOC-2 (Differentially Expressed In Ovarian Carcinoma 2).The following year, using a mouse macrophage cell line, Xu et al. (2) cloned the cDNA encoding the p96 isoform (and a smaller splice variant p67) of a protein homologous to the *Drosophila* *disabled* with high similarity to the human DOC-2. Human DOC-2 gene was thoroughly characterized in 1996 by Albertsen et al. (3). The name Disabled-2 (Dab2/DAB2) was first introduced by Xu et al. (4). Since the early days of its cloning, DAB2 emerged as a cytosolic adaptor with important roles in the regulation of endocytosis and signal transduction due to its multiple protein binding motifs. DAB2 participates in the formation of multiprotein complexes involved in clathrin-mediated endocytosis (CME) where it assists with recognition and recruitment of receptors to clathrin-coated pits. DAB2 selection of protein cargo inside clathrin pits occurs via its phosphotyrosine‐binding (PTB) domain which binds to NPVY motif found in the various receptors, such as LDL receptor (5), TGFβ receptor (6), and vascular growth factor (VEGF) receptor (7). Furthermore, interaction with carboxy-terminal tail of myosin VI is required for intracellular transport of clathrin-coated vesicles (8). DAB2 has been implicated in several receptor-mediated signaling cascades and related physiologic functions, such as cell adhesion, differentiation and angiogenesis. DAB2 p96 also interacts directly with phosphoinositides and binds both clathrin and the α-adaptin subunit of AP2 (5). The shorter p67 isoform of DAB2 lacks a region with two DPF motifs, one NPF motif and the two clathrin boxes, therefore it does not interact with AP2 or clathrin (**Figure 1**) and plays a minor role in endocytosis (9). DAB2 has also been described as a putative tumor suppressor, as it was found downregulated in a variety of epithelial cancers (10). Dab2 expression is highly regulated and some of the mechanisms reported include gene promoter hypermethylation (10), regulation by microRNAs (11), translational regulation via Dab2 transcript interaction with hnRNPE1 (12), and protein phosphorylation on multiple serine residues (13).

## DAB2 and TGFβ Signaling

TGFβ signaling plays an essential role in immune tolerance in both the innate and adaptive arm of the immune response. TGFβ acts on all immune cells and among its effects, it suppresses effector function, proliferation and promotes the generation of FoxP3^+^ regulatory T cells (Tregs) (14). TGFβ1 gene ablation in mice results in multi-organ autoimmunity and death (15). Activation of TGFβ signaling is initiated by binding of the cytokine to TGFβ receptor II (TGFBR2), its association with TGFBR1 (Alk5), and formation of a receptor heterotetramer. Upon ligand binding, TGFBR1 phosphorylates Smad2 and Smad3 which form a complex with Smad4 and enter the nucleus to regulate gene expression (16). TGFβ also signals through a non-canonical pathway, which leads to MAPK activation and is particularly important during epithelial to mesenchymal transition (EMT) (17). TGFBR complex is internalized in clathrin-coated vesicles (CCV) or within lipid rafts/caveolae domains. After internalization, TGFβ receptor subunits are localized in early endosomes followed by their recycling back to the cell surface. The proper activation of Smad signaling cascade is documented to require an intact clathrin mediated endocytosis, while lipid-rafts are required for MAPK activation (18). TGFβ canonical signaling pathway is believed to mediate immune regulation and was found dysregulated during inflammation (19, 20).

In non-immune cells, DAB2 was shown to associate with TGFBR1 and TGFBR2, direct them to CCV, and permit the subsequent Smad2/3 activation (6). DAB2 also plays a role in TGFBR recycling from early to recycling endosomes (21). While enhancing TGFβ canonical pathway, DAB2 is thought to divert TGFBR from the lipid rafts and to restrict MAPK-dependent alternative pathway (22). Given the involvement of DAB2 in the TGFβ canonical signaling pathway in a variety of studies, it was expected that DAB2 modulates this pathway in immune cells and to be part of the complex machinery in immune tolerance mediated by TGFβ. While this remains open question in some cells of the immune system, recent work from our lab suggested at least in dendritic cells (DCs), this is not the case and that DAB2 is dispensable for TGFβ-induced Smad2/3 activation (23).

## DAB2 Expression in Immune Cells and its Role in Inflammatory Response

Since the initial description of DAB2 expression in macrophages (4) and later reports on the role of DAB2 in this immune lineage, Dab2 mRNA and/or protein expression has been described and analyzed in T cells and DCs. We will discuss the available evidence for DAB2 roles in these cells in the following sections. The overall emerging picture of DAB2 in the immune system is that of a negative regulator of the immune response and inflammation, although an exemption to that rule has also been demonstrated in neuroinflammation. Elevated Dab2 expression in lesional macrophages/microglia correlated with the severity of autoimmune encephalomyelitis in mice and its deficiency reduced the neuroinflammatory symptoms (24). As null Dab2 knockout in mice is embryonically lethal (25), the current understanding of Dab2 participation in immune responses comes from studies using Dab2 silencing in cell lines or mice with conditional deletion of Dab2.

### DAB2 in Antigen Presenting Cells

Antigen presenting cells (APCs) provide important aspects of the innate immune response by secreting inflammatory mediators and are the crucial players in activating T cell-mediated, antigen-speciﬁc adaptive immune responses. After phagocytosis, APCs process the antigens, conjugate them to MHCI or MHCII, and display these complexes on their surface to CD8^+^ and CD4^+^ T lymphocytes, respectively. Activation of APCs results in an increased expression of costimulatory molecules (CD80, CD86, CD40) and secretion of soluble mediators that act synergistically to trigger a powerful sequence of events to promote T cell activation, expansion, effector function, and the eventual pathogen or cancer cell elimination. The extent and the inflammatory phenotype acquired by T cells after activation depends on the nature and the strength of the signal provided by APCs. For example, Th1-type responses occur when APCs release IL-12, which in turn stimulates the differentiating CD4^+^ T cells to produce TNFα and IFNγ, while the Th17 effector CD4+ T cells producing IL-17A differentiate in the presence of IL-6 and TGFβ (26). While macrophages are equipped to mount an immune response to mediate pathogen killing and process antigen to the already primed CD4^+^ T cells, DCs are more efficient in priming naïve CD4^+^ T cells.

DAB2 is highly expressed in macrophages and DCs at steady state and dramatically decreased following cell activation by microbial antigens. This decrease may impact their ability to phagocytose, process antigen and present antigens to T cells. The exact subcellular localization of DAB2 expressed on APCs cells has not been characterized in detail but the expression of the p96 isoform suggests its association with membrane domains as it was reported for normal and transformed epithelial cells. The distribution of DAB2 protein in distinct puncta in murine macrophages and DCs (23, 27) resembles that observed in fibroblast and platelets (28, 29). However, contrary to epithelial cells, DAB2 in DCs did not appear to colocalize with the endosomal compartments (23). In this review, we will synthesize the current findings on DAB2 modulation in immune cells and its functional implications as summarized in **Table 1**.

**Table 1:** Summary of Dab2 expression in immune cells, its effects on their immune function and implications for inflammatory outcomes.

Cell type	Function	Implication
**CD4^+^FoxP3^+^ Treg**	Enhances gap junction between Treg and T responder cells	Promotes protective function of Tregs in experimental model of colitis (30)
**Progenitor and mature BMDM**	Enhances cellular adhesion and spreading/anti-proliferative	Overexpression mediates the promotion of a myeloproliferative syndrome in ICSBP KO mice (31)
**Murine myeloid cells**	-	Protects from liver inflammation in LDLR^−/−^ fed with HFD (32)
**Murine BMDM**	Favors macrophage polarization toward M2 phenotype	Protects liver and lung from inflammation in murine model of endotoxemia (27)
		Dampens inflammatory cytokines in adipose tissue and protects from insulin-resistance in HFD mice (27)
	Favors iNOS expression	Aggravates MS severity in murine model of EAE (24)
**Human macrophages in BAL**	Favors M2 phenotype (27)	-
**Murine peritoneal** **Macrophages**	Inhibits nitric oxide, inflammatory cytokines and chemokines	Mediates the anti-inflammatory effect of BRP on macrophages (33)
**RAW 264.7** **(Murine macrophages)**	Inhibits MyD88-dependent TLR signaling (27)	-
	Inhibits TRIF-dependent TLR signaling (34)	-
**Rat brain infiltrating myeloid cells**	-	Dab2^+^ myeloid cells in the brain correlates with inflammation induced by cryoinjury (35)
**Human monocyte–derived DCs**	Suppresses CD40, CD83, CD86, TNF, and IL-12p70	Regulates inflammation in patients with active VKH (36)
**Murine BMDCs**	Suppresses activation markers, inflammatory cytokines and migration	Inhibits efficacy of vaccine therapy against tumor (37)
**Murine BMDCs**	Supresses IL-12, IL-6, and T cell proliferation	Mediates immunoregulatory effects of quercetin on murine model of atherosclerosis (38)
**DC2.4** **(murine DCs)**	Supresses IlL23a, IL-1β and CD40	Mediates protection by DC2.4 during intestinal inflammation in mice (23)

HFD, high fat diet fed mice; BRP, Brazilian Red Propolis; MS, multiple sclerosis; EAE, experimental autoimmune encephalomyelitis; BAL, bronchoalveolar lavage; BMDM, bone marrow–derived macrophages; DCs, dendritic cells; BMDC, bone marrow–derived DCs; -, when not described.

### Macrophages

The original cloning of the full-length murine Dab2 cDNA was done with SV40 transformed murine macrophage cell line by Xu et al. (4) In 2002, Rosenbauer et al., demonstrated the expression of DAB2 in bone marrow–derived macrophages (BMDM) differentiated *in vitro* with colony stimulating factor 1 (CSF-1) (31). This work also provided the first description of negative regulation of DAB2 expression by an inflammatory stimulus (IFNγ). The authors showed that interferon consensus sequence binding protein (ICSBP, now interferon regulatory factor 8 or IRF8) restricts basal expression of Dab2 in macrophages and mediated the inhibitory effects of IRF8 on Dab2 gene transcription and protein expression in both myeloid progenitors and mature monocytes. Moreover, following stimulation of ICSBP/IRF8^−/−^ BMDM with CSF-1, DAB2 p96 protein was upregulated, phosphorylated, accumulated in the cytoskeleton/membrane fractions, and promoted BMDM adhesion and spreading (31). Given the importance of IFNγ/IRF8 axis for proliferation and differentiation of myeloid cells (39), DAB2 repression via this pathway suggested that DAB2 influences these very processes. Indeed, overexpression of DAB2 had anti-proliferative effects on BMDM culture and promoted macrophage adhesion and spreading, both prominent features observed in myeloproliferative syndrome in IRF8^−/−^ mice (31). Interestingly, IFNγ/IRF8 axis also promotes elimination of intracellular bacteria through the oxidative burst (40), although the role of DAB2 as an IFNγ-regulated gene has not been studied in this context yet.

DAB2 expression in macrophages was addressed in the context of neuroinflammation. Moon et al. (35) described high expression of DAB2 in myeloid cells infiltrating the brain during inflammation caused by cryoinjury in rats, and contrasted it with low expression in neurons and glial cells in homeostasis. Peripheral macrophages are known to infiltrate the brain parenchyma concurrently with the activation of brain-resident microglia even before the development of neurological symptoms of experimental autoimmune encephalomyelitis (EAE), promoting neuroinflammation at early stages of the disease (41). DAB2^+^CD11b^+^ myeloid cells were found recruited to the spinal cord during acute phase of EAE in mice immunized with MOG_35-55_ peptide (24) or with myelin basic protein (MPB) (42), followed by a decline during the recovery phase in both studies. The same pattern was observed in the compressive spinal cord injury in rats (43), indicating an association between DAB2-expressing myeloid cells recruitment and neuronal inflammation in laboratory rodents. DAB2 p96, but not the p67 variant, was found in its phosphorylated form during early stage of inflammation in MBP-induced EAE, indicating protein activation by inflammatory mediators (42). Of note, considering the absence of a good marker to distinguish monocytes recruited from the periphery from microglia, it is likely that the high staining for DAB2 in CD11b^+^ cells indicated upregulation of DAB2 expression in microglia during neuroinflammation. DAB2 expression was also upregulated in astrocytes in the inflamed spinal cord, suggesting that neuronal cells can also respond to inflammatory stimuli by upregulating DAB2 (24, 42).

To address the putative causative role of DAB2 in myeloid cells in neuroinflammation, Jokubaitis et al. (24) studied DAB2 expression in human MS and in EAE induced in wild-type and Dab2 conditional knockout mice. The authors showed the DAB2 expression within the early active MS lesions (defined by the presence of macrophages with MOG-positive myelin debris coinciding with significant axonal injury) was very high and declined in late acute and further in chronic active lesions. They also evaluated the role of DAB2 in the pathogenesis of neuroinflammation in murine MOG-induced EAE. To address this while overcoming the problem of embryonic mortality of Dab2 null knockouts, the authors crossed Dab2^fl/fl^ mice with Meox2-Cre mice to generate animals in which Dab2 was deleted from the embryo, while preserving Dab2 expression in the extra-embryonic tissue. EAE severity was reduced in heterozygous and homozygous conditional knockouts, with improved survival, reduced disease score, axonal injury, and iNOS expression in the spinal cord (24). Dab2-deficient BMDM stimulated with LPS expressed dramatically lower level of iNOS mRNA expression, showing macrophages modulation toward an inflammatory phenotype via DAB2. Another finding from this study that distinguished it from previous work with Tregs and BMDM was that DAB2 expression was diminished in response to all-*trans* retinoic acid and TGFβ_1,_ while it was not affected by IFNγ or LPS (24). These discrepancies remain unresolved. Since DAB2 was also upregulated in astrocytes, more specific models of tissue specific deletion of Dab2 would be needed to precisely address the role of this protein in peripheral macrophages or microglia. Nonetheless, those reported findings may be of relevance for the understanding of DAB2 role in human MS.

The first description of DAB2 in human macrophages was in a report by Dawson et al. (44), which described Dab2 mRNA and protein expression in macrophages derived from human monocyte cell line (THP-1) stimulated with a PKC inducer, phorbol 12-myristate 13-acetate (PMA), as well as in primary human monocyte-derived macrophages (HMDM). The pathways responsible for DAB2 expression in THP-1 macrophages were not elucidated in this study, but its PKC-dependent phosphorylation is likely involved due the nature of this cell model. DAB2 p96 and p67 in human macrophages were associated with the expression of its partner AP-2 (α2 subunit), and the two colocalized in the cell periphery in a punctate staining pattern. Dab2 silencing showed that DAB2 was not involved in the internalization of LDL (via LDR receptor, LDLR) or oxLDL (via scavenger receptors) by human macrophages (44). As macrophages play a decisive role in atherosclerotic lesion progression, the role of myeloid DAB2 in atherosclerosis has been further tested in LDLR^−/−^ mice reconstituted with myeloid Dab2-deficient bone marrow (BM) from Dab2^fl/fl^ LysM-Cre mice fed with high fat diet (HFD) (32). Mice that received Dab2-deficient BM showed an exacerbated hepatic inflammation during chronic HFD, characterized by increased TNFα, IL-6 and IL-1β, extensive inflammatory infiltrate and decreased levels of lipids in the liver and serum, suggesting a prominent participation of myeloid-DAB2 in regulating hepatic response to high-fat diet. A reduction in atherosclerotic lesion burden was observed as a secondary effect to the liver damage (32). Hypercholesterolemia promotes monocytosis and macrophages differentiation via granulocyte-macrophage colony-stimulating factor (GM-CSF) (45). Interestingly, the atherosclerotic plaques from mice transferred with Dab2-deficient BM, despite a reduced hypercholesteremia, harbored increased numbers of macrophages and apoptotic cells, indicating the absence of DAB2 promoted the recruitment/accumulation of macrophages into the plaques (32). It suggested that these myeloid cells maybe more sensitive to GM-CSF or another inflammatory stimulus and that DAB2 expression in myeloid cells may render them less inflammatory. However, the phenotype/activation state of the macrophages accumulated in the plaque lesions has not been addressed in this study.

Macrophage polarization is a reversible process by which macrophages assume distinct molecular and functional phenotypes as a reaction to specific microenvironmental stimuli. Macrophages can be polarized into classically activated (M1) and alternatively activated (M2) cells, and the latter can be further divided into M2a, M2b, M2c, and M2d subcategories. These macrophages differ in their cell surface markers, secreted cytokines and biological functions. M1 macrophages are generally considered as pro-inflammatory, assisting with pathogen phagocytosis and secretion of pro-inflammatory cytokines and microbicidal molecules, while M2 macrophages are thought to contribute to the resolution phase of inflammation and tissue repair (46). The role of DAB2 in macrophage polarization has been addressed in an excellent and comprehensive study by Adamson et al. (27) DAB2 p96 and p67 were found repressed in BMDM and RAW264.7 cells under M1 polarizing condition (IFNγ+LPS) but upregulated in M2 macrophages (IL-4). DAB2 is also likely to play a role in polarization of human macrophages, as DAB2 was highly expressed in human M2 (CD14^+^CD163^+^) in comparison to M1 (CD14^+^CD163^-^) macrophages from bronchoalveolar lavage (27). IFNγ, LPS (TLR4 ligand), or LTA (lipotechoic acid, TLR2 agonist) used individually significantly downregulated Dab2 mRNA and protein, while M2-promoting (IL-4) and tolerogenic signals (IL-10, TGFβ, retinoic acid) upregulated it in RAW264.7 macrophages (27). This suggested that DAB2 repression could play a role in promoting the inflammatory phenotype acquired by macrophages after exposure to M1-polarizing signals. The same study, using siRNA-mediated Dab2 knockdown in RAW 264.7 macrophages or BMDM from Dab2^fl/fl^ LysM-Cre mice, showed that loss of DAB2 potentiated the inflammatory response of macrophages to LPS or LTA as assessed by IL-1β, Ptgs2 (COX-2), IL-6, and TNFα. Consistent with these *in vitro* observations, Dab2^fl/fl^ LysM-Cre mice fared poorly in a model of endotoxemia than their WT controls, with worsened clinical score and higher expression of IL-6 and TNFα in the liver and lungs (27).

Under M1 polarizing conditions, DAB2-deficient macrophages expressed significantly higher levels of M1-associated transcripts for IL-1β, TNFα, IL-6, and CCL2. Moreover, in a phenotype switching experiment, DAB2 deficiency significantly enhanced the propensity of M2 macrophages to switch into an M1 phenotype but inhibited the switch from M1 into M2 phenotype (27).

In atherosclerotic plaques formation, M1 macrophage polarization promotes inflammation in the adipose tissue while M2 macrophages are protective (47). In HFD-fed mice with chronic inflammation, DAB2 expression in myeloid cells favored the shift of adipose tissue-macrophages into M2 phenotype to the detriment of M1, and protected mice from insulin-resistance associated from adipose inflammation to a small degree. The levels of serum IL-6, an M1 cytokine, were also lower in DAB2-expressing macrophages indicating DAB2 protective effects in systemic inflammatory responses (27). Therefore, at the current stage of research, the expression of DAB2 appears to favor the macrophage shift toward an M2 phenotype, promoting anti-inflammatory and a pro-resolving phenotype in mouse macrophages and in selected murine models of inflammation.

The molecular mechanisms by which DAB2 restricts the inflammatory profile in macrophages have been addressed by studies that demonstrated a pivotal role of DAB2 in inhibiting myeloid differentiation factor 88 (MyD88)- or TIR-domain-containing adapter protein-inducing interferon-B (TRIF)-dependent signaling after TLR activation (27, 34). The NF-κB and MAPK signaling pathways are activated by virtually all TLRs and are involved in driving M1 polarization in macrophages. Both pathways are controlled by TRAF6 interacting with cell surface cytokine and pathogen-associated molecular pattern (PAMP) receptors. DAB2 was shown to physically interact with TRAF6 via two domains (see **Figure 1**) and this interaction was critical for downstream inhibition of phosphorylation of the p65 subunit of the NF-κB complex at Ser^536^, IKKβ phosphorylation on Ser^177^, and ultimately reduced nuclear translocation of the NF-κB complex and reduced NF-κB-dependent gene transcription in macrophages (27). DAB2 also negatively controlled p38 MAPK activation.

DAB2-deficiency potentiated the macrophage response to LPS (TLR4 ligand, that signal via both MyD88 and TRIF adaptor proteins) and to LTA (ligand for TLR2 which utilizes MyD88 but not TRIF) (27). Although TRAF6 was initially described as necessary for in signal transmission in MyD88- but not TRIF-dependent pathway (48), other studies showed that TRAF6 is recruited to TRIF and receptor-interacting protein kinase (RIPK)1 kinase, which in turn activates TGF-β-activated kinase 1 (TAK1) during inflammatory response (49–51). Interestingly, Hung et al. (34) suggested that in RAW264.7 macrophages stimulated with LPS followed by microarray gene expression profiling, DAB2 was mainly involved in regulation of TRIF-dependent genes, with only a modest effect on MyD88-dependent genes. As examples, DAB2 repressed TRIF-dependent IFNβ and RANTES gene expression in LPS-treated RAW 264.7 without affecting Myd88-dependent TNFα expression (34). Contrary to Adamson et al. (27), but in support of the predominant effect of DAB2 on the TRIF-dependent pathway, Hung et al. (34) observed no effect of DAB2 on NF-kB or MAPK activation. This discrepancy remains unresolved, and perhaps may be ascribed to the differences in experimental approaches to DAB2 silencing and/or knockout. For example, transduction with a lentiviral vector could potentially overstimulate the TRIF-dependent TLR-signaling in macrophages and lead to overinterpretation of the significance of the DAB2 involvement in TRIF-dependent pathway.

Currently available data is not sufficient to pinpoint the exact point of overlap between DAB2 and the branches of TLR signaling pathways in macrophages. It was proposed that DAB2 functions as a negative regulator of clathrin-dependent LPS-induced TLR4 internalization into endosomes. According to the “clathrin sponge” model, DAB2 may complex with clathrin in the steady state, inhibiting its association with TLR4. Upon LPS exposure and DAB2 downregulation, that complex is disrupted and clathrin is left free to interact with TLR4 promoting its endocytosis and TRIF-mediated signaling (34). In support of that hypothetical scenario, DAB2 p96 phosphorylation was observed in macrophages during TLR4 internalization induced by LPS (34), a post-transcriptional modification known for inhibiting DAB2 interaction with clathrin (52).

Regardless of the current controversies regarding the precise mechanism of DAB2 involvement, the emerging role of DAB2 as a novel negative regulator of innate immune response in macrophages is well supported by the existing data. Future work will need to address not only the precise molecular mechanisms, but also investigate them in the context of inflammatory microenvironment, varying stimuli, and target macrophage cell populations. Of note, M2 macrophages may promote tumor growth (53), and DAB2^+^ tumor associated macrophages have shown to play central role in lung metastasis formation (54), therefore would be of interest to address the role of DAB2 in myeloid cells during tumor progression. Another unexplored aspect of DAB2 biology is the mechanisms of downregulation in activated macrophages. DAB2 expression may be reciprocally regulated by NF-κB in macrophages. Bueno-Silva et al. (55) have shown an upregulation of DAB2 in peritoneal macrophages treated with Brazilian Red Propolis (BRP) during LPS-mediated activation. BRP has been described as a non-specific NF-κB inhibitor with anti-inflammatory effects in macrophages (33).

#### Dendritic Cells

As a bridge between innate and adaptive immune responses, DCs balance anti-inflammatory and pro-inflammatory responses. Immature DCs help maintain immune tolerance, while mature DCs facilitate and promote the immune response. Published findings pointed out to a positive correlation between DAB2 expression and DCs differentiation, and a repression of DAB2 during DCs activation/maturation. Microarray analysis in murine bone marrow–derived DCs (BMDCs) showed that DAB2 was signiﬁcantly induced during BMDCs differentiation with GM-CSF (37). Although the transcriptional signature of BMDCs differentiated under this protocol does not fully correspond to that found in primary murine DCs (56), such approach remains valuable to understand the significance of DAB2 for DCs differentiation. DAB2 p96 isoform was also detected in murine splenic DCs and in human monocyte-derived DC (hMoDC) (23, 37), suggesting that DAB2 may contribute to *in vivo* DCs differentiation and/or their immunogenicity. STAT5, which is essential for GM-CSF-mediated DCs differentiation, was required for GM-CSF-induced DAB2 expression in BMDCs. Also, DAB2 expression in differentiating DCs required activation of PI3K/AKT/hnRNP E1pathway (37). Under normal conditions, hnRNP E1 (heterogeneous nuclear ribonucleoprotein E1; or PCBP1, Poly(rC)-binding protein 1) inhibits Dab2 translation by blocking the TGFβ-activated translation (BAT) element in the 3′-untranslated region of Dab2 mRNA transcript (12). Phosphorylation of hnRNP E1 (e.g., by protein kinase B PKB/Akt2 downstream of TGFβ activation) induces its release from the BAT element and allows for unimpeded translation of Dab2 mRNAs. Similar mechanism of “translational release” during DC differentiation with GM-CSF did not correlate with activation of TGFβ signaling (37). During GM-CSF-induced differentiation, DAB2 negatively controlled their inflammatory phenotype, dampening the expression of activation markers CD86, CD80, CD40 and MHCII, as well as their phagocytic and migratory ability. As an effect, Dab2-knockdown DCs were more effective in stimulating antigen-specific cytotoxic CD8 T cells responses in mice and enhanced the efficacy of DC-based tumor immunotherapy than wild-type DCs (37).

A study which investigated the atheroprotective effects of quercetin in mice showed that DAB2 expression in DCs correlated with the protection offered by this flavonol against atherosclerotic lesions in ApoE^−/−^ mice fed a HFD (38). Treatment with quercetin increased DAB2 expression, suppressed LPS-induced maturation of DCs in a dose-dependent manner, inhibiting the secretion of pro-inflammatory cytokines and expression of activation markers induced by LPS, as well as DC-induced T cell proliferation. Quercetin-mediated inhibition of LPS-induced IL-12, IL-6, and T cell proliferation required DAB2 upregulation in DCs. Mechanistically, promotion of immune tolerance by DAB2 in quercetin-treated DCs occurred via inhibition of Src/PI3K/AKT pathway and consequent suppression of NF-κB activation (38). DAB2 has been shown to bind the Src homology 3 domain of c-Src, reduce its phosphorylation at Tyr^416^ and maintain it in an inactive conformation in prostate epithelial cells (57). It appears that this mechanism is also functional in BMDCs as silencing Dab2 transcript elevated pTyr^416^-Src levels (38). Quercetin exerts anti-inflammatory and anti-oxidative effects in a variety of immune cells, perhaps most frequently studied in macrophages, where it inhibits M1 polarization, iNOS, and IL-12 expression (58). It is tempting to speculate that its anti-inflammatory effects in macrophages are mediated by DAB2 restoration since Src/PI3K/Akt/NF-κB signaling pathway similar to that described in BMDCs, since this pathway is equally important in macrophage activation (59).

More recently, our group has refined the role of DAB2 expression in DCs, by stably knocking out Dab2 in murine immortalized DCs, DC2.4, using CRISPR-CAS9 system (23). This approach allowed us to better determine the effects of DAB2 on the immune function of DCs after activation, ruling out any differences that may arise from Dab2 ablation on DCs differentiation. We observed that DAB2 contributed to an immune regulatory (immature) phenotype in DCs after activation by LPS, through promotion of phagocytic activity, inhibition of IL-1β and IL-23a, as well as CD40 expression (23). We hypothesized that inhibition of Il-1β and Il-23a may be related to the autophagy in DCs, a process known to counter-regulate transcription of IL1β and consequently IL-23a in human and mouse APCs (60, 61). Indeed, we found that Dab2 was to some extent permissive for autophagy and protected DC2.4 cells from staurosporin-induced apoptosis (23). These findings are in contrast to those described for epithelial cells, where DAB2 inhibited Beclin-1-Vps34 induction of autophagy and promoted cell death (62).

Finally, we showed that DAB2 expression in DCs is relevant for their function *in vivo*. Among DCs, DAB2 was expressed at the highest levels in CD103^-^CD11b^+^ intestinal DCs (23), a population responsible for the induction of Th17 and Th1 inflammatory responses in the gut (63). Colitis induced by the adoptive transfer of naïve CD4^+^CD45RB^high^ T cells into Rag2^−/−^ mice correlated with a reduction in DAB2 levels in CD11b^+^ DC in the colonic lamina propria upon, suggesting that DAB2 plays a role in keeping the tolerogenic profile in intestinal DCs during homeostasis, and its downregulation may contribute to exacerbated immune responses in inflammatory bowel diseases (IBDs). Further, a variety of TLR ligands downregulated DAB2 expression *in vitro* in BMDCs differentiated in a protocol that yields a population of DCs similar in composition and phenotype to mouse intestinal DCs (iBMDC), in a mixed Myd88/TRIF-dependent manner (23). These findings suggest that the exposure to microbial products is the main driver of DAB2 downregulation in DCs in the inflamed colon, consistent with the high bacterial load in this intestinal segment and the impaired epithelial barrier function in IBD. Different from what was described for macrophages, DAB2 repression in LPS-activated iBMDC was shown to be a biphasic event, where the rapid downregulation of DAB2 protein precedes a prolonged inhibition at the mRNA level, suggesting an intricate mechanism to ensure a quick and sustained DAB2 repression in activated DCs (23). Downregulation of DAB2 in DCs may not be limited to PAMPs but may also be compounded by the effect of the host’s inflammatory mediators. As we discussed earlier, Dab2 it has been identified as a IFNγ-responsive gene in macrophages (31) and increased mucosal IFNγ is a hallmark of Crohn’s disease and experimental murine colitis (64). Importantly, consistent with the hypothesis that DAB2 restricts DC immunogenicity, we showed that adoptive transfer of Dab2-deficient DC exacerbated experimental colitis in mice (23).

As discussed above, expression of DAB2 is the highest among CD11b^+^CD103^−^ iBMDCs as intestinal lamina propria DC. DAB2 PTB domain interacts with NPXY motifs in integrin β1 (65) and is required for β1 integrin endocytosis and focal adhesion disassembly, necessary for cell migration (66). It is thus tempting to speculate that other integrins may be similarly regulated by DAB2. However, human and mouse CD11b and ITGAE/CD103 proteins lack NPXY motif as an endocytic sorting signal, which makes it less likely that DAB2 is directly involved in their endocytic trafficking. However, this motif is present in the C-terminus of integrin beta 7 (ITGB7), with forms the functional heterodimer αEβ7 with CD103. It is plausible, though not yet tested, that DAB2 interaction with αEβ7 regulates its cell surface expression, which could in turn impair the otherwise tolerogenic effects of CD103^+^ DCs in the gut mucosa. Contrary to findings described in non-immune cells, but in agreement with work with macrophages, we found that DAB2 did not control the canonical TGFβ signaling in DCs (23). It is plausible, that as in macrophages, the regulatory effects of DAB2 on DCs immune activation are related to DAB2 interaction with components of inflammatory cascades triggered during cell activation and their ultimate suppression.

**Figure 2:**
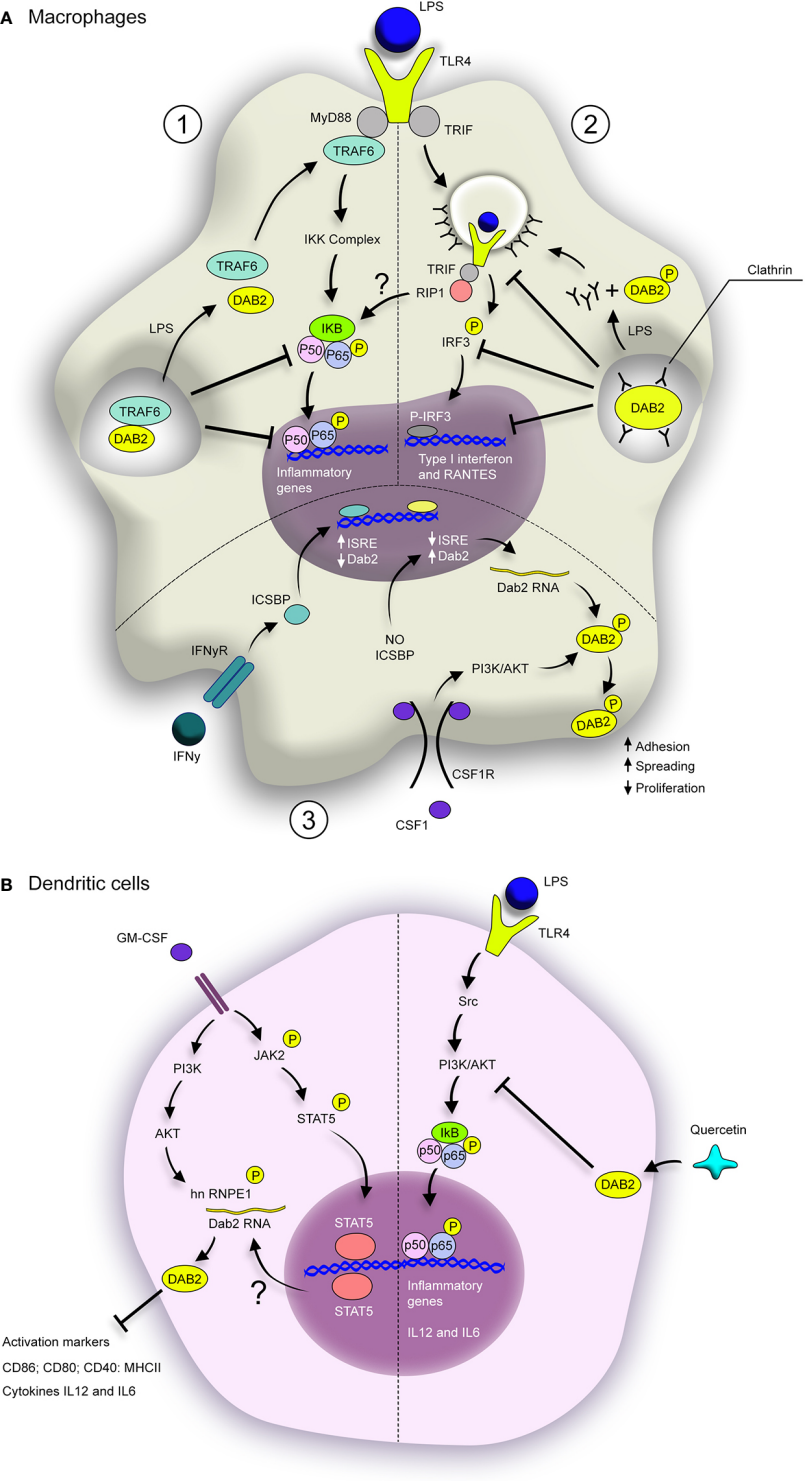
Dab2 modulates inflammatory pathways in macrophages and dendritic cells. **(A)** DAB2 modulates MyD88-dependent (1) and TRIF-dependent TLR signaling in macrophages (2), as well as their response to CSF-1 (3). (1) DAB2 direct interaction with TRAF-6 inhibits the downstream cascade that leads to NF-κB translocation to the nucleus and induction of inflammatory genes. (2) DAB2 binding to clathrin inhibits TLR4 loading into endosomes and triggering of TRIF-mediated IRF3 activation, necessary to induce type I interferon and RANTES. Stimulation of TLR4 by LPS triggers the dissociation of DAB2 from TRAF6 (1) or clathrin (2) thus promoting these signaling pathways. (3) Dab2 transcription is repressed by IFNγ/ICSBP during macrophage differentiation with CSF-1, but absence of ICSBP promotes DAB2 expression. In this context, CSF-1 induces DAB2 phosphorylation, accumulation at the cell surface and promotion of adhesion and spreading, as well as inhibition of proliferation. **(B)** GM-CSF-induced differentiation of BMDCs promotes DAB2 expression in a STAT5- and PI3K/AKT-dependent fashion. hnRNP E1 phosphorylation by AKT enhances DAB2 protein levels, which inhibits expression of activation markers and inflammatory cytokines. TLR4 activation in DCs induces NF-κB activation via Src/PI3K/AKT pathway. Quercetin dampens this pathway by stimulating DAB2 expression in LPS-treated BMDCs, which inhibits the induction of inflammatory cytokines, likely through direct interaction between DAB2 and c-Src. BMDCs, bone marrow–derived dendritic cells; ISRE, IFN-stimulated response elements; ICSBP, interferon consensus sequence binding protein.

The DAB2 has been studied in humans DCs in the context of systemic autoimmunity directed against tissues expressing melanosome-associated tissues called Vogt-Koyanagi-Haranda (VKH) (36). The reported findings suggest that DAB2 repression in DCs may indeed contribute to pathogenesis of autoimmune diseases. Dab2 mRNA and protein were found decreased in monocyte-derived DCs from patients with active VKH, and restored to normal levels after immunosuppressive therapy (36). Epigenetic modifications in Dab2 gene were found in DCs from VKH patients and may contribute to its repression during this disease. Additionally, Dab2 overexpression in DCs from active VKH patients led to decreased expression of activation markers CD83, CD40 and CD86, as well as production of TNFα and IL12p70 after LPS challenge, which ultimately rendered DCs less efficient in inducing differentiation of naïve CD4^+^ T cells into Th1 and Th17 effector cells (36). In effects, this observation showed that restoration of DAB2 expression in DCs counter-regulated their inflammatory phenotype. These findings provide further support to the notion that DAB2 provided an important checkpoint in DCs and protects from exacerbated inflammation in various disease settings. A cartoon depiction of the known mechanisms by which DAB2 plays an anti-inflammatory roles in APC, as well as what is known about its modulation in these cells are provided in **Figure 2**.

### DAB2 in T Cells

Relatively little is known about DAB2 expression, regulation, and function in T lymphocytes. The report by Jain et al. (30) showed that among T cells, Dab2 mRNA is restricted to peripheral FoxP3^+^CD4^+^ Tregs and to mature FOXP3^+^CD4^+^CD8^−^ T cells in the thymus. ChIP analysis identified a FoxP3 consensus binding site in the 5’-untranslated region - 2.3-kb upstream of the transcriptional initiation site of the Dab2 gene, indicating Dab2 as a potential target gene of FOXP3 (30). Dab2 mRNA was also induced during FOXP3^+^CD4^+^ Tregs differentiated *in vitro* with TGFβ, TGFβ and all-trans retinoic acid (ATRA), but not with ATRA alone. These studies focused on Dab2 transcript and did not address DAB2 protein expression in Tregs. Under homeostatic conditions, mice with T-cell specific deletion of Dab2 were healthy, with no signs of autoimmunity. The frequency of thymic and peripheral CD4^+^FOXP3^+^ cells was unchanged in Dab2 conditional knockout mice when compared to controls. Dab2-deficient Tregs were also phenotypically indistinguishable from wild-type Tregs in that they expressed normal levels of CTLA-4, and IL-10. GITR and IL-7R were also apparently not altered, though the data were not shown (30). However, when tested in the *in vitro* suppression assay, Dab2-deficient Tregs entirely lost their ability to suppress proliferation of responder T cells. Since DAB2 was previously shown to interact with connexins that make up the gap junctions (67) and since the immunosuppressive effects of Tregs on effector T cell proliferation is in part mediated by cAMP transfer between the two cells via gap junctions, Jain et al. (30) tested whether Dab2 deficiency impaired this process. Indeed, Dab2-deficient Tregs had elevated intracellular level of cAMP (consistent with its build-up) and less efficiently transferred a gap junction tracer compound calcein-AM to the co-cultured responder T cells during *in vitro* immunosuppression assay. Jain et al. (30) further tested immunosuppressive capacity of Dab2-deficienct Tregs *in vivo*. To accomplish this, wild type or Dab2^−/−^ Tregs were co-transferred along with CD4^+^CD25^−^ T cells into Rag1^−/−^ mice and the development of chronic colitis was monitored. Although the authors concluded that “Dab2-deficient T cells “were as effective as WT Treg cells in preventing the induction of colitis”, the report provided limited data to support this observation. Another variation of this experiment in the same paper, this time with Treg co-transferred with naïve CD4^+^CD25^-^CD45RB^high^ T cells, showed that transfer of Dab2^−/−^ Tregs into mice with an established colitis was not effective in reducing the severity of colitis. Admittedly, the analysis of these models was somewhat rudimentary, and the authors did not reconcile the observed differences. While the picture of DAB2 in the function of Tregs is far from complete, it appears that under homeostatic conditions, DAB2 is not necessary for the maintenance of Tregs (at least as tested to date) but may be required for their immunosuppressive functions under inflammatory stress. The roles of DAB2 in promoting Treg function need to be more thoroughly studied, including their ability to produce other soluble mediators and their expression of regulatory cell surface molecules such as CD39, CD73, LAG-3, and PD-1. It is also plausible that DAB2 plays distinct roles in thymic and inducible/peripheral Tregs and that its role may vary in different microenvironments that imprint specific functional and phenotypic signature in Tregs.

## Conclusions and Future Directions

Limited studies published to date have shown that DAB2 contributes to the immune regulatory phenotype in FoxP3^+^ Tregs, macrophages, and DCs. Thus, approaches to preserve or promote DAB2 expression in immune cells may represent attractive therapeutic strategy in chronic inflammatory and certain autoimmune conditions. This generalization has to be interpreted with caution as DAB2 was shown to promote inflammatory responses in autoimmune encephalomyelitis and spinal cord injury. Therefore, the precise role of DAB2 in inflammatory and autoimmune diseases still remains poorly characterized. There is need for better understanding of DAB2 regulation and its roles in different immune cells, their specialized sub-populations, and their responses under specific inflammatory conditions.

## Author Contributions

All authors contributed to the article and approved the submitted version.

## Funding

NIH 5R01 DK109711 (FG and PK) and PANDA Endowment in Autoimmune Diseases (PK).

## Conflict of Interest

The authors declare that the research was conducted in the absence of any commercial or financial relationships that could be construed as a potential conflict of interest.
